# *Cymbopogon Citratus* Functionalized Green Synthesis of CuO-Nanoparticles: Novel Prospects as Antibacterial and Antibiofilm Agents

**DOI:** 10.3390/biom10020169

**Published:** 2020-01-22

**Authors:** Tijo Cherian, Khursheed Ali, Quaiser Saquib, Mohammad Faisal, Rizwan Wahab, Javed Musarrat

**Affiliations:** 1 Faculty of Agricultural Sciences, Department of Agricultural Microbiology, Aligarh Muslim University, Uttar Pradesh, Aligarh 202002, India; tvarghese891@gmail.com (T.C.); khursheedamu@gmail.com (K.A.); musarratj1@yahoo.com (J.M.); 2 Zoology Department, College of Sciences, King Saud University, P.O. Box 2455, Riyadh 11451, Saudi Arabia; rizwannano@gmail.com; 3 Department of Botany & Microbiology, College of Sciences, King Saud University, P.O. Box 2455, Riyadh 11451, Saudi Arabia; faisalm15@yahoo.com; 4 School of Biosciences and Biotechnology, Baba Ghulam Shah Badshah University, Rajouri 185234, Jammu & Kashmir, India

**Keywords:** *Cymbopogon citratus*, copper oxide nanoparticles, esters functionalization, antibiofilm, gas chromatography-mass spectrometry

## Abstract

Chemically synthesized copper oxide nanoparticles (CuONPs) involve the generation of toxic products, which narrowed its biological application. Hence, we have developed a one-pot, green method for CuONP production employing the leaf extract of *Cymbopogon citratus* (CLE). Gas chromatography-mass spectrometry (GC-MS) analysis confirmed the capping of CuONPs by CLE esters (CLE-CuONPs). Fourier-transform infrared (FTIR) showed phenolics, sugars, and proteins mediated nucleation and stability of CLE-CuONPs. X-ray diffraction (XRD) and transmission electron microscopy (TEM) revealed CLE-CuONPs between 11.4 to 14.5 nm. *Staphylococcus aureus*-1 (MRSA-1), *Staphylococcus aureus*-2 (MSSA-2) exposed to CLE-CuONPs (1500 µg/mL) showed 51.4%, 32.41% survival, while *Escherichia coli*-336 (*E. coli*-336) exposed to 1000 µg/mL CLE-CuONPs showed 45.27% survival. Scanning electron microscopy (SEM) of CLE-CuONPs treated *E. coli*-336, MSSA-2 and MRSA-1 showed morphological deformations. The biofilm production by *E. coli*-336 and MRSA-1 also declined to 33.0 ± 3.2% and 49.0 ± 3.1% at 2000 µg/mL of CLE-CuONPs. Atomic absorption spectroscopy (AAS) showed 22.80 ± 2.6%, 19.2 ± 4.2%, and 16.2 ± 3.6% accumulation of Cu^2+^ in *E. coli*-336, MSSA-2, and MRSA-1. Overall, the data exhibited excellent antibacterial and antibiofilm efficacies of esters functionalized CLE-CuONPs, indicating its putative application as a novel nano-antibiotic against multi drug resistance (MDR) pathogenic clinical isolates.

## 1. Introduction

The popular choice for nanoparticles (NPs) synthesis include the chemical and physical methods. However, the utilization of toxic chemicals in the above processes narrowed their wider applications. Consequently, the green method was developed for the synthesis of NPs using the biological approach. In this context, the manipulation of nucleation and growth stages of NPs synthesis deserves special attention because it helped the nanotechnologists to produce varying types of NPs [1]. Therefore, researchers have shown enormous interest towards the synthesis of inorganic and organic NPs [2,3], enabling them to be used in a range of biomedical settings [4,5].

The capping agents, reducing agents, as well as the reaction solvents were considered important for the NPs synthesis via the green approach. Particularly, the crude leaf extracts from different plants showed the presence of bio-active molecules including the proteins [6], polysaccharides [7], polyphenols [8], terpenoids [9], and esters [10]. These agents played an important role as reducing and capping entities in the synthesis of metallic NPs.

The metal oxide NPs, owing to their fascinating physicochemical characteristics of transition, have received considerable attention in the field of nanotechnology. Amongst such metal oxides, copper oxide (CuO) is a p-type semiconductor with a narrow band gap of 1.2–1.7 eV. CuO is widely acclaimed for its photoconduction relevance, fabrication of various electro-optics devices [11,12,13], high temperature conductive superconductors [14], and gas sensors [15]. The crystalline monoclinic structures of CuONPs have engrossed great usability in a range of scientific and technical applications [16]. The applicability of CuONPs as antimicrobials [17], pesticidal formulations [18], dye photo-degradation [19], drug delivery and imaging agent has revolutionized the field of medico-diagnostics. CuONPs have also been explored as an efficient recyclable catalyst [20,21]. Traditionally, CuONPs have been synthesized by the physical and chemical methods, including solid state thermal decomposition [22], chemical precipitation [23], sol gel [24], and pulsed wire explosion methods [25]. All methods do have a common drawback to generate toxic products, which are incongruous for the biological utilization. As a safer alternative, the biological schemes employing plants leaf extracts superseded the chemical methods [9,10,13].

*Cymbopogon citratus* (CLE) is an aromatic plant belonging to the family Gramineae [26]. The active phytochemicals present in CLE are long chain hydrocarbons, alcohols, ketones, esters, and aldehydes. Essential oil mainly consists of terpenes and triterpenoids. Major flavonoids include luteolin and its 6-C and 7-*O*-glycosides, isoorientin 2′-*O*-rhamnoside, quercetin, kaempferol, and apigenin. The phenolic compounds in CLE were primarily found to be elimicin, catecol, chlorogenic acid, caffeic acid, and hydroquinone [26]. Studies have indicated the successful use of *Cymbopogon* extracts for the fabrication of metallic NPs like silver, gold, copper, cerium dioxide, which have demonstrated their antibiosis and dye degradation benefits [27,28,29,30]. The large applications of CuONPs, as well as the bio-actives of CLE, have led us to scrutinize the crude aqueous extract of CLE as a reducing, capping and stabilizing agent for the bio-synthesis of CLE-CuONPs. To the best of our knowledge, until now, there is no comprehensive and systematic report available, which has demonstrated that the three bio-active esters of CLE (dipropyleneglycol-1 diacrylate (C_12_H_18_O_5_), α-monoolein (C_21_H_40_O_4_), and Isooctyl phthalate (C_24_H_38_O_4_) actively reduce Cu^2+^ to CuONPs. The present work is novel, and the first of its kind on CLE-CuONPs, demonstrating its antibacterial and antibiofilm efficacies against multi drug resistance (MDR) clinical isolates. Hence, this study aimed to (i) optimize the synthesis of CuONPs using CLE extracts, (ii) characterization of biogenically synthesized CLE-CuONPs, (iii) analysis of bio-active compounds of CLE-CuONPs, and (iv) antibacterial and antibiofilm potential of CLE-CuONPs against clinical isolates.

## 2. Materials and Methods

### 2.1. Chemicals and Bacterial Strains

Copper sulphate (CuSO_4_·5H_2_O), hydrochloric acid (HCl), sodium hydroxide (NaOH) were of highest analytical grade purchased from Hi-media, Pvt. Ltd. Mumbai, India. Glassware were procured from Borosil, India. Before experiments, all glassware was treated with 1 N H_2_SO_4_, and thoroughly washed with tap water followed by rewashing with deionized ultrapure water (TKA Genpure, Niederelbert, Rheinland-Pfalz, Germany). The Gram-positive methicillin-resistant *Staphylococcus aureus*-1 (MRSA-1), methicillin-sensitive *Staphylococcus aureus*-2 (MSSA-2), and Gram-negative extended spectrum *β*-lactamases (*ESβL*) producing *Escherichia coli*-336 (*E. coli*-336) clinical isolates was a kind gift from the Department of Microbiology, Jawaharlal Nehru Medical College, Aligarh Muslim University, Aligarh, India. After the in-house validation, the clinical isolates were sub-cultured in Luria–Bertani (LB) culture medium (Hi-media, Pvt. Ltd. Mumbai, India) at 37 °C in a shaker (180 rpm) for overnight, and stored at −20 °C in 20% glycerol for long-term preservation.

### 2.2. Preparation of Cymbopogon Citratus (CLE) Extract

The preparation of *Cymbopogon citratus* leaves extract (CLE) were carried out by following our earlier described procedure [10]. Precisely, 50 g green leaves were collected from forest cover range at Kottayam district (9.4710933°N, 76.7650384°E) Kerala, India. Sample identification was done by taxonomist in the Department of Botany, Aligarh Muslim University, India. The leaves were washed with ultrapure water Milli Q (MQ) water thoroughly and minced to fine green paste by the use of mixer grinder. Paste was then added to 100 mL of water, and kept for 20 min at ambient temperature. The liquid extract of CLE was then filtered through Whatman paper No.1 and stored at −20 °C till further use.

### 2.3. Synthesis Optimization and Characterization of CLE-CuO NPs

Synthesis and optimization of CLE-CuONPs yield were investigated by the previously described method [9]. Briefly, CLE-CuONPs formation was assessed under the steady elevation of CLE concentrations (10, 20, 30, 40 and 50%) and different pH (4.0, 6.0, 8.0 10.0 and 12.0), while keeping the CuSO_4_ concentration constant (0.25 mM). pH adjustment was done by enriching the reaction mixtures with 0.1 M HCl and NaOH. The suspensions were kept at 60 °C for 3 h under constant stirring. A dry greyish black powder of CLE-CuONPs was finally obtained by centrifugation at 3000 rpm for 5 min, followed by vacuum filtration, and drying for 4 h at 80 °C. The physicochemical properties of CLE-CuONPs were determined using the following techniques. Surface plasmon resonance (SPR) of CLE-CuONPs was recorded by the use of double beam UV-Vis spectrophotometer (Cintra 101, GBC Scientific Equipment Ltd., Dandenong, VIC, Australia) in the range of 540–660 nm. SPR peaks at different percentages of CLE and pH were recorded. For the blank, ultrapure water water was used. Background absorption was subtracted from the reading of CLE [9]. Powdered sample of CLE-CuONPs was used for the X-ray diffraction (XRD) patterns (Rigaku Corporation, Tokyo, Japan) at 40 kV, 30 mA current, and CuKα radiation (k = 1.54 Å). Intensities were recorded between 20–80° 2θ angles. CLE-CuONPs size and crystalline phase purity were calculated using the Debye–Scherrer’s equation: D = 0.9λ/βcosθ [9]. Structural analysis of CLE-CuONPs was done using the scanning electron microscope (SEM) at 20 kV (JEOL Ltd., Tokyo, Japan). Fine powder of CLE-CuONPs was placed on the carbon tape, followed by gold coating using vacuum sputter. The percentage of C, O and Cu elements in CLE-CuONPs was analyzed using energy dispersive X-ray (EDS) spectrum (Oxford Instruments INCA x-sight, Concord, MA, USA) [9]. CLE-CuONPs was further characterized using the transmission electron microscope (TEM) (JEOL, Tokyo, Japan) at an accelerating voltage of 200 kV. Samples were prepared by dispensing 10 µL of CLE-CuONPs on to the Cu grid, and the liquid was vacuum dried (80 °C, 6 h) [9].

### 2.4. GC-MS and FTIR Analyses of CLE-CuONPs

GC-MS analysis of CLE and colloidal CLE-CuONPs solution was performed on GC-MS (GC-7890A, Agilent Technologies, Santa Clara, CA, USA) equipped with paraphernalia such as silica capillary column (30 m length, 0.25 mm inner diameter, 0.25 µm film thickness), a triple-axis detector, and oven (60–325 ˚C at 5 ˚C/min for 5 min). The inlet and interface temperatures were set as 280 ˚C and 325 ˚C, respectively. Helium (He) was used as carrier at 1.0 mL/min flow rate. In addition, 1 µL of the sample was injected under a split of 20:1. EIMS: electron energy, 70 eV. The MS data were deciphered using 62,000 patterns compiled as an NIST database library [9]. Similarly, in order to confirm CLE bio-actives capping on NPs, CLE alone and CLE-CuONPs were analyzed by Fourier-transform infrared (FTIR) (Perkin Elmer, Shelton, CT, USA). For FTIR, CLE and CLE-CuONPs were separately mixed with spectroscopic grade of KBr in a ratio of 1:100, followed by the recording of spectra at a resolution of 4 cm^−1^ [31].

### 2.5. Antibacterial Activities

#### 2.5.1. Well Diffusion Assay, MIC, and MBC Determinations

The antibacterial activities of CLE-CuONPs were assessed by a well diffusion assay following our previously described method [31]. Briefly, the overnight grown cultures of *E. coli*-336, MRSA-1, and MSSA-2 (OD_600_ = 0.1 ~ 10^7^ colony forming unit (CFU)/mL) were used for the study. Cultures were spread on LB agar plates, wells of 6 mm diameter were cut and filled with CLE alone (as control), and varying concentrations of CLE-CuONPs (500, 1000, 1500 and 2000 μg/mL) were added in respective wells. All plates were then incubated for 24 h at 37 °C followed by the analysis of zone of inhibition [31]. For the minimum inhibitory concentration (MIC) and minimum bactericidal concentrations (MBC), the overnight grown bacterial cultures (~10^7^ CFU/mL) in liquid LB media containing 500, 1000, 1500, 2000, 2500 and 3000 μg/mL of CLE-CuONPs were incubated for 24 h at 37 °C. An aliquot of 100 μL from the cultures was spread on LB agar plates, followed by incubating the plates at 37 °C for 24 h. MIC and MBC were determined based on the colony forming ability of the test strains.

#### 2.5.2. Effect of CLE-CuONPs on Growth and Viability of Bacteria

The growth and viability tests were performed according to our previously described method [31]. In brief, 20 μL of overnight grown cultures *E. coli*-336, MRSA-1 and MSSA-2 were seeded into 96-wells of microtiter plates. LB medium containing fixed volume (300 μL) of CLE-CuONPs 125, 250, 500, 1000, and 2000 μg/mL were added to the respective wells. Untreated bacteria were used as control in all experiments. At the time intervals of 2 h, changes in the optical density was recorded at 620 nm (OD_620 nm_) using a microplate reader (Thermo Scientific Multiskan, China). The percent viability was also estimated by comparing the OD_620 nm_ of CLE-CuONPs treated bacterial cultures with untreated control experiments after 24 h.

#### 2.5.3. SEM Imaging of Bacteria and NPs Interaction

Morphological changes in the bacterial cells were done by following our previously described method [10]. In brief, *E. coli*-336 and MRSA-1 cells were treated with CLE-CuONPs (1000 μg/mL) for 24 h at 37 °C. Under the similar condition, *E. coli*-336 and MRSA-1 not exposed to CLE-CuONPs were taken as control. After the incubation, the bacterial cells were spun at 3000 rpm for 5 min, and placed in 2.5% glutaraldehyde solution for 4 h. Bacterial cells were dehydrated in an ascending series of 30, 50, 70 and 90 and 100% ethanol. Finally, 100 μL bacterial cells were evenly spread on the clean glass cover, and deposited on SEM stub after sputter coated with gold-palladium. Morphological analysis was done at 15 kV.

#### 2.5.4. Intracellular Uptake of CLE-CuONPs

The uptake of CLE-CuONPs in bacterial cells were analyzed by our previously described method [9]. Quantitative estimation was done employing the atomic absorption spectroscopy (AAS) of *E. coli*-336, MSSA-2, and MRSA-1 grown for 24 h in the presence of CLE-CuONPs (1000 μg/mL). Under the identical conditions, a separate set of above strains were grown without CLE-CuONPs, which were taken as control cells. After 24 h, the bacterial cells were spun down at 3000 rpm for 15 min. Washing of pellets were done twice to remove surface bound NPs. Pellets were then subjected to digestion in aqua regia (HNO_3_:HCl = 1:3 *v*/*v*) at 60 °C. The digests were diluted to 100 mL in ultrapure deionized water, and finally analyzed on a double beam AAS (GBC Model 932B plus, GBC Scientific Equipment Ltd., Dandenong, VIC, Australia). The amount of internalized CLE-CuONPs was quantified by comparing the concentration of Cu^2+^ in the treated cells, with the amount of treatment concentration (1000 µg/mL). In parallel, the internalization propensity of CLE-CuONPs was qualitatively evaluated on TEM following our previous method [9]. *E. coli*-336 and MRSA-1 were treated with CLE-CuONPs (500–1000 µg/mL) for 24 h at 37 °C. Under the similar conditions, *E. coli*-336 and MRSA-1 grown without CLE-CuONPs were taken as control. After 24 h, cells were centrifuged at 3000 rpm for 15 min and washed twice. Bacterial cells were fixed using glutaraldehyde (2.5%), osmium tetraoxide (1%), and then embedded in Epon resin (Polybed 812). Ultra-thin sections were prepared, and staining was done using uranyl acetate and lead citrate (Sigma Aldrich, St. Louis, MO, USA). Image analysis was done on TEM (100/120 kV) [9].

#### 2.5.5. Effect of CLE-CuONPs on Bacterial Biofilm

Antibiofilm efficacy of CLE-CuONPs were evaluated against the three test strains by standard crystal violet (CV) assay, as described in our earlier study [9]. Precisely, 100 μL of *E. coli*-336, MSSA-2, and MRSA-1 grown for overnight in LB culture medium (~10^7^ cells/mL) were seeded into 96-wells of microtiter plate (Corning, NY, USA). A fixed volume (200 µL) of culture medium, containing varying concentrations (125–2000 μg/mL) of CLE-CuONPs were added to the respective wells. Untreated bacterial cells in LB culture medium were used as parallel positive controls. Plates were allowed to dry at 37 °C for 24 h. The loosely adhered cell suspensions were removed by washing with autoclaved phosphate buffer saline (PBS). All wells were then dispensed with 200 µL of CV solution (0.25%), and incubated for 30 min at 37 °C. The unbound CV were washed, and wells were dried at room temperature. CV bounded to the bacterial cells were finally dissolved in 200 μL of ethanol (95%), and the absorbance was read at OD_620 nm_ on a microplate reader. Additionally, CLE-CuONPs induced biofilm eradication was also investigated by confocal laser scanning microscopy (CLSM), following our previously described method [9]. Briefly, *E. coli*-336 and MRSA-1 were grown on glass cover, and treated with sub-lethal concentrations of CLE-CuONPs (500 and 1000 μg/mL) for 24 h. The biofilms adhered on the glass cover were first immersed in PBS to remove the planktonic cells, and then in 1 μM concanavalin-A-fluorescein isothiocyanate (Con-A FITC). Con-A FITC binds specifically to sugars, glycoproteins, and glycolipids accumulated in the extracellular polysaccharides (EPS) layers around the bacterial cells. The reduction in the biofilm was visualized by change in the fluorescence intensity of Con-A FITC (488–530 nm) on CLSM (Leica TCS SP5, Leica Microsystems, Mannheim, Germany) [9].

### 2.6. Statistics

The statistical analyses were done on Sigma Plot 11.0 (Sigma Plot 11.0, Systat Software, Inc., San Jose, CA, USA). A Holm–Sidak test for multiple comparisons versus a control group was used for one-way analysis of variance (ANOVA). The data were expressed as mean ± SD of at least three independent experiments done in triplicate. The differences were considered statistically significant if * *p* < 0.05 unless otherwise stated.

## 3. Results and Discussion

### 3.1. Synthesis and Optimization of CLE-CuONPs

**Figure 1:**
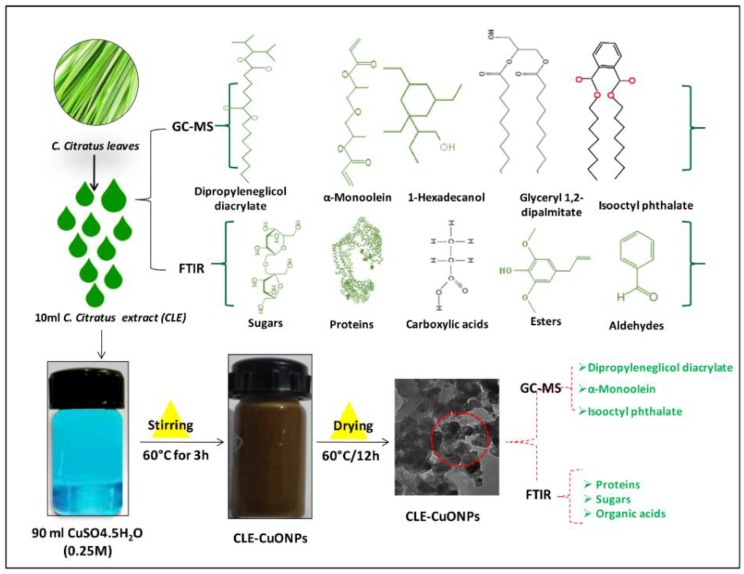
Graphical representation of CLE-CuONPs synthesis.

**Figure 2:**
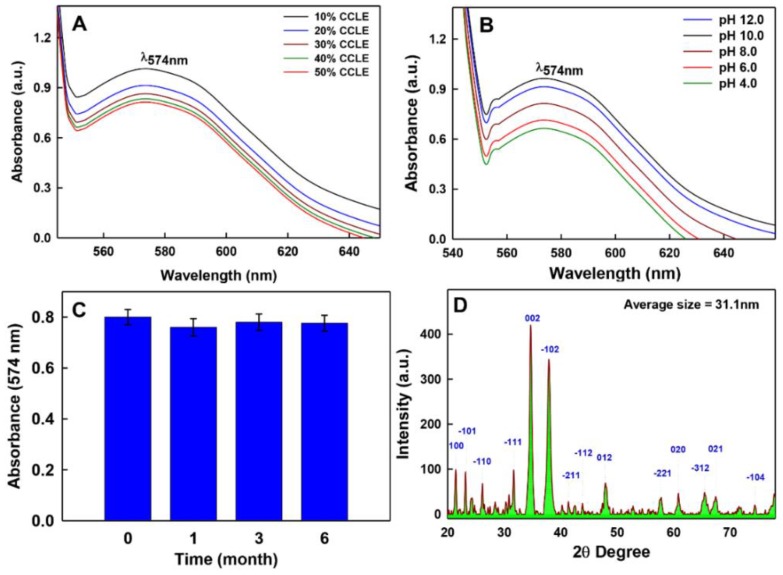
Physicochemical characterization of CLE-CuONPs. UV-Vis spectra-based optimization of CLE-CuONPs synthesis as a function of CLE concentration (10, 20, 30, 40 and 50%) (**A**), and pH (4.0, 6.0, 8.0, 10.0 and 12.0) (**B**) showing SPR peak at ~574 nm. (**C**) shows the stability of CLE-CuONPs based on the SPR measurements up to six months (error bars represent the mean ± SDSE of three replicates); (**D**) demonstrates X-ray diffraction (XRD) patterns of biologically synthesized CLE-CuONPs.

The yield of bio-inspired CLE-CuONPs was assessed by monitoring the characteristic UV-visible absorbance band in the range of 540-650 nm under varied concentrations of CLE (10, 20, 30, 40, and 50% *v*/*v*), and pH (4.0, 6.0, 8.0, 10.0, and 12.0) of the reaction mixture. After 3 h of CLE exposure to CuSO_4_ at 60 °C, CLE-CuONPs formation was marked with the change of reaction mixture color from green to greyish black (Figure 1). Such change has been related with the excitation of SPR or inter-band transitions, thus reflecting the metallic nature of NPs [32]. The CLE-CuONPs SPR peak at 574 nm was very close to an SPR peak of ascorbic acid-CuONPs at 550 nm [33]. We have observed a hypochromic shift (i.e., a decrease in absorption from 9.3 to 8.2) in SPR at 574 nm upon increasing CLE concentration from 10 to 50%, while the concentration of CuSO_4_ throughout the reaction was fixed at 0.25 mM. This hypochromic shift in SPR peak indicates the reduction in NP size [34], which highlights the fact that an increase in CLE bio-actives enhanced the Cu^2+^ reduction, nucleation, and capping kinetics, respectively (Figure 2A). In case of pH manipulation, while keeping the CLE (10%) and CuSO_4_ (0.25 mM) concentrations fixed (Figure 2B), the UV-vis spectra exhibited hyperchromic shift (i.e., an increase in SPR absorption as 6.0, 6.2, 7.4 and 9.2) reflecting an increase in the size of NPs at pH 4.0, 6.0, 8.0, and 10.0, respectively. However, an increase in pH from 10.0 to 12.0 caused hypochromic shift in SPR peak, marking the optimum reduction of Cu^2+^ to CLE-CuONPs at pH 10.0. The stability of colloidal reaction mixture of CLE-CuONPs was measured using UV-Vis spectrophotometric measurments. The analysis showed peak at 574 nm for 6 months, further confirming the stability of synthesized NPs with no precipitation (Figure 2C). The rationale for CLE-CuONPs synthesis was based on the fact that 10% CLE and pH 10.0 showed significant long-term colloidal dispersion and stability. Such characteristics could be due to the soft corona of CLE bio-actives induced steric hindrance around the particles. These effects may reduce the aggregation of NPs due to the electrostatic interactions [9].

### 3.2. Morphology and Crystallinity of CLE-CuONPs

**Figure 3:**
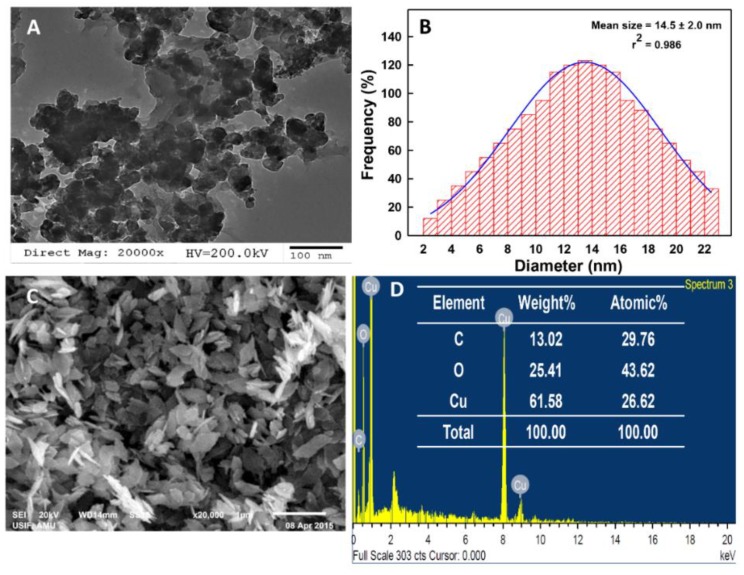
Electron microscopic analyses of CLE-CuONPs. (**A**) demonstrates TEM images of CLE-CuONPs; (**B**) depicts the particle size distribution in TEM images; (**C**) shows scanning electron microscope (SEM) micrographs of CLE-CuONPs and (**D**) is the energy dispersive X-ray (EDS) spectrum showing the percentage of C, O and Cu elements in CLE-CuONPs.

The XRD data further suggested the crystalline nature of CLE-CuONPs prepared with 10% CLE at pH 10.0 (Figure 2D). The diffraction peaks obtained at 2θ values 32.69°, 34.68°, 37.87°, 47.93°, 57.88°, 60.87°, 65.42°, 67.45°, and 74.50° corresponds to 110, 002/−111, 111/200, 112, 021, −113, 022, 311, and 004/−222 hkl lattice planes, respectively. The XRD peaks were readily indexed to the monoclinic symmetry of CuO with lattice constants a = 4.684 Å, b = 3.425 Å, c = 5.129 Å, and β = 99.47 Å obtained by following the International Centre for Diffraction Data (ICDD, obtained from JCPDS 080-1268) [35]. The average crystal size of CLE-CuO-NPs was determined to be 31.1 nm. In addition, the data obtained using the TEM and SEM analyses confirmed the morphology of NPs synthesized using aqueous CLE (10%) at pH 10.0. The TEM micrograph exhibits that CLE bio-actives significantly contributed in the bio-reduction of Cu^2+^ or Cu^3+^ into crystalline CLE-CuONPs (Figure 3A). The average crystal size of 14.5 ± 2.0 nm (Figure 3B) was calculated by processing the TEM image of Figure 3A, employing the ImageJ multidimensional image processing software. It is also apparent that, with our optimal synthesis conditions, CLE bio-actives could configure nascent NPs into noticeable different morphologies as spherical, hexagonal and oval shapes (Figure 3A). In addition, varying NPs sizes (2 to 22 nm) were generated, although the majority of NPs ranged between 12 to 14 nm (Figure 3B). On the other hand, the SEM image (Figure 3C) exhibits a bud or needle like morphology. The formation of needle-like CuONPs is a well observed phenomenon of the aggregation of small NPs, and monoclinic crystalline nature of CuO [36,37]. EDS analysis further indicated the presence of elemental copper (26.62%) and oxygen (43.62) in association with carbon (29.76%) (Figure 3D), which can be assigned to the corona of CLE bio-actives moieties adsorbed onto the CuONPs.

### 3.3. Cu^2+^ Reduction Mechanism

**Figure 4:**
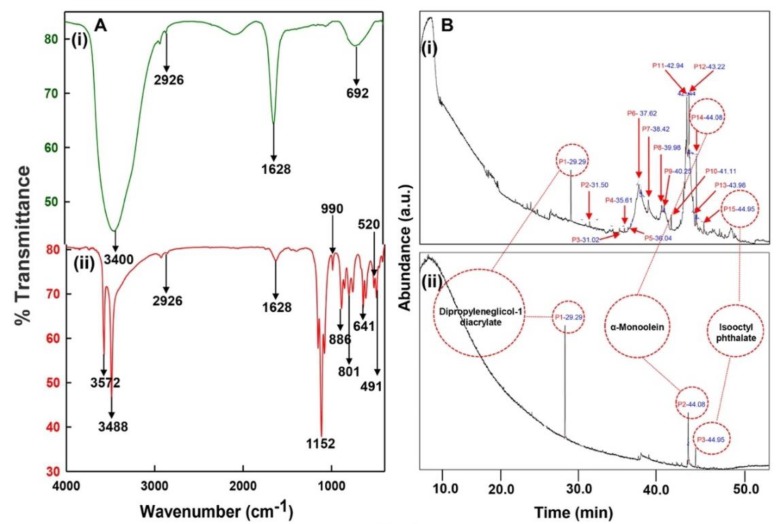
FTIR and GC-MS analyses of CLE and CLE-CuONPs. (**A**) comparison of FTIR spectra of CLE (spectrum-i) and CLE-CuONPs (spectrum-ii); (**B**) GC-MS analysis (spectrum-i) shows a typical chromatogram of CLE indicating 15 peaks of different bio-actives. Spectrum-ii shows three CLE compounds: dipropyleneglycol-1 diacrylate, α-monoolein, and iso-octyl phthalate associated with CLE-CuONPs.

The organic support of CLE bio-actives in the reduction of Cu^2+^ and capping of CuONPs was ascertained by comparing the FTIR spectrum of CLE-alone (Figure 4A(i)) and CLE-CuONPs (Figure 4A(ii)). The spectrum of CLE-CuONPs (Figure 4A(ii)) shows that the appearance of sharp bands at 3572, 3488 and 2926 cm^−1^ are likely due to stretching of -OH, -NH and CHO groups associated with the skeletons of CLE bio-actives [38]. Changes in the sharp peak at 1628 cm^−1^ in the CLE-CuONPs spectrum justify the role of -C=O stretch of ester and amide groups in NP formation (Figure 4A(i,ii)) [39]. Similarly, the formation of a series of sharp peaks between 1152 and 611 cm^−1^ was described in Supplementary Data (Table S1). The peaks that were assigned to -CN and C-O-C groups indicate the viable role of protein moieties in the synthesis of NPs [40]. Importantly, the banding and stretching vibrations between 520 and 491 cm^−1^ are typically known to reflect the signature of bonds between metal and oxygen [41]. In view of the reducing and ligation properties of esters in CLE, it can be articulated that the oxygenated functional groups of bio-actives orchestrated an energy-efficient reduction and nucleation of Cu^2+^ into CuONPs.

### 3.4. GC-MS Analysis of CLE-CuONPs

The GC-MS shows 15 different peaks (P1-P15) for 11 types of bio-actives esters (Figure 4B(i)) in aqueous extract of CLE (Table 1). The structure of esters is the association of cyclic/hemicyclic ring and a linear hydrocarbon molecule bearing various oxygenated functional groups (Table 1). Hence, it can be speculated that a single CLE ester may produce two or even multiple spectral signatures with different retention times and peak areas. The mass and charge ratio (*m*/*z*) of esters were used to identify the compound with NIST retention index library [42,43]. The heteroatom-functionalized long-chain hydrocarbons have been known to play important roles in controlling the morphology, size, and monodispersity of NPs. The presence of such bio-active hydrocarbons, aldehyde, and long chain fatty acids in CLE has also been evidenced in methanol extract of CLE leaves [44]. GC-MS analysis of CLE-CuONPs revealed the appearance of three bio-active esters including Dipropyleneglicol diacrylate (P1), α-Monoolein (P2) and Isooctyl phthalate (P3) (Table 2). These bio-active esters reflect their plausible role in the reduction and nucleation of Cu^2+^ or Cu^3+^ into CLE-CuONPs (Table 1). Di-isooctyl phthalate has also been reported in the extract fractions of *Limonium bicolour* [45].

### 3.5. Antibacterial Effects of CLE-CuONPs

#### 3.5.1. Antibacterial Activity, MIC and MBC Determination

**Figure 5:**
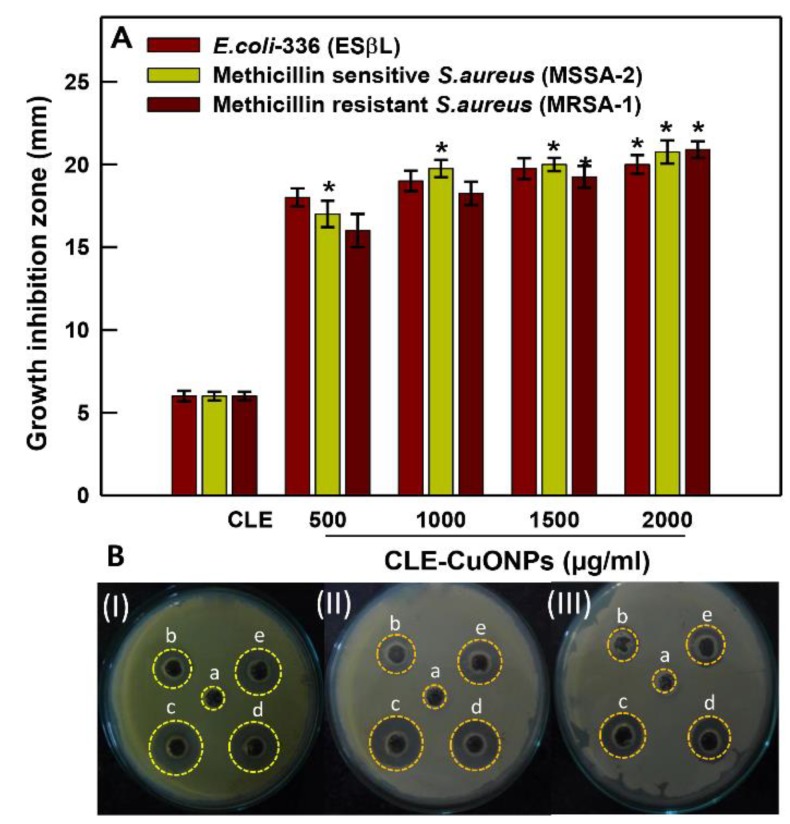
Antibacterial activity assessment. (**A**) shows assessment of antibacterial activity of CLE alone and CLE-CuONPs by a well diffusion assay. A volume of 100 μL CLE (10%) was taken as control. Each histogram represents the mean ± SD of two independent experiments done in triplicate; (**B**) representative culture plates of (i) Gram-negative *ESβL* producing *E. coli*-336, (ii) Gram-positive methicillin-sensitive MSSA-2, and (iii) and methicillin-resistant MRSA-1 exhibiting zone of growth inhibition at (a) 10% CLE, and (b) 500, (c) 1000, (d) 1500 and, (e) 2000 μg/mL of CLE-CuONPs after 24 h of exposure.

Histograms shown in Figure 5A demonstrate a dose dependent reduction in the growth of clinical isolates. *E. coli*-336 showed increase in the zone of inhibition, measured as 12.0 ± 0.5 to 20.25 ± 0.6 mm at 500 to 2000 µg/mL of CLE-CuONPs (Figure 5A,B). The observed changes are in line with antibacterial effects of green synthesized CuONPs against *E. coli* cells [46]. Comparatively, MSSA-2 showed a higher zone of inhibition measured as 10.7 ± 0.8 to 18.25 ± 0.7 mm, while MRSA-1 showed 9.2 ± 1.0 to 16.2 ± 0.5 mm zone of inhibition after exposure to CLE-CuONPs (500 to 2000 µg/mL) (Figure 5A,B). CLE-alone diluted to 1:100 (*v*/*v*) has produced non-impressive cytotoxic effects (Figure 5B(I–III)). The antibacterial activities of CLE-CuONPs tested against the clinical isolates can be hierarchized as *E. coli*-336 > MSSA-2 > MRSA-1. Moreover, a similar trend of antibacterial effects was observed while determining the MIC and MBC of CLE-CuONPs against the test strains (Table 3). The MIC and MBC values of CLE-CuONPs against *E. coli*-336 were found to be 500 and 1500 µg/mL. MSSA-2 showed the MIC and MBC at 1000 and 2000 µg/mL, while 1500 and 2500 µg/mL of CLE-CuONPs were found to be effective as MIC and MBC in MRSA-1 (Table 3). Overall, our data on the antimicrobial effects of CLE-CuONPs supported well with the inherent properties of copper based antimicrobial formulations against MDR superbug MRSA [47].

#### 3.5.2. Comparative Planktonic Growth Inhibition Assessment

**Figure 6:**
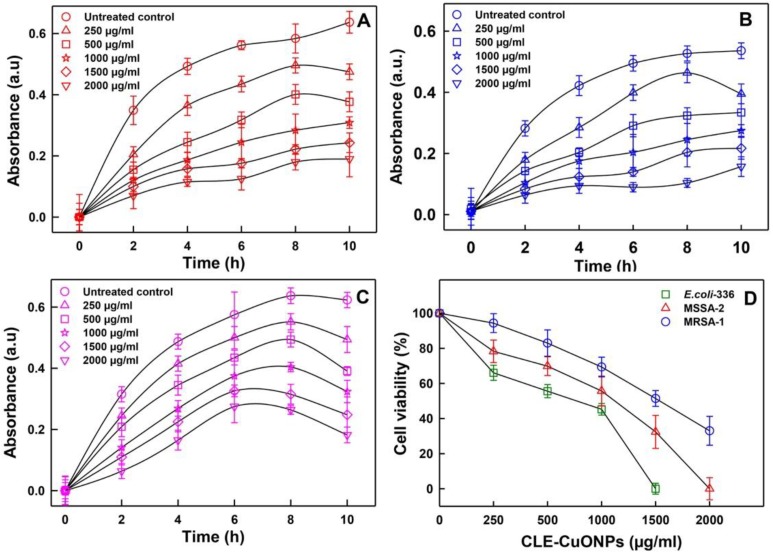
Antibacterial activity of CLE-CuONPs against MDR bacterial growth. Changes in the absorbance (OD_620 nm_) of clinical bacterial isolates at increasing CLE-CuONPs concentrations (**A**–**C**). (**A**) Gram-negative *ESβL* producing *E. coli*-336; (**B**) Gram-positive methicillin-sensitive MSSA-2, and (**C**) methicillin-resistant MRSA-1; (**D**) shows reduction in percent cell viability of clinical isolates treated with 250, 500, 1000, 1500 and 2000 μg/mL of CLE-CuONPs.

A dose and time dependent reduction in the proliferative yield (OD_620 nm_) of *E. coli*-336, MSSA-2, and MRSA-1 was recorded (Figure 6A–C). *E. coli*-336 cells after 10 h incubation with CLE-CuONPs (250–2000 µg/mL) showed OD_620 nm_ in the range of 0.47 to 0.19, which are significantly lesser than the OD_620 nm_ of 0.53 recorded for the untreated control (Figure 6A).

**Figure 7:**
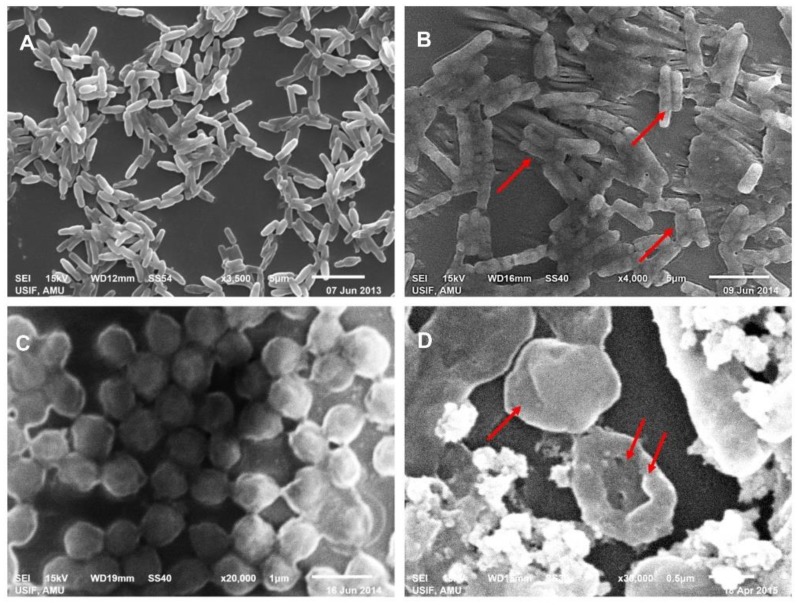
Ultrastructural analysis showing interaction of CLE-CuONPs with bacterial cells. Representative SEM images showing the cellular damage and surface binding of CLE-CuONPs with (**B**) *E. coli*-336 and (**D**) MRSA-1. The images in (**A**,**C**) show the untreated controls of *E. coli*-336, and MRSA-1.

CLE-CuONPs also affected the growth of MSSA-2 and MRSA-1. Changes in the OD_620 nm_ values recorded between 0.39 to 0.15, and 0.49 to 0.18, as compared to the values of 0.53 and 0.62 in untreated controls (Figure 6B,C). Furthermore, the antibacterial effect of CLE-CuONPs suspensions (250–2000 µg/mL) on the percent survival of *E. coli*-336, MSSA-2 and MRSA-1 were estimated after 24 h of exposure. Figure 6D exhibits an apparent decline in the percent survival of *E. coli*-336 to 45.27 ± 3.3% at 1000 µg/mL of CLE-CuONPs, whereas, *E. coli*-336 exposed to MBC (1500 µg/mL), its survival was reduced to zero. MSSA-2 and MRSA-1 at the sub-lethal concentration (1500 µg/mL) of CLE-CuONPs exhibited 32.41 ± 9.4% and 51.4 ± 4.5% survival. At 2000 µg/mL, MSSA-2 survival decreased by almost 99.9% signifying its MBC value. At the same concentration, MRSA-1 survival declined to 33.0 ± 8.2%. The growth inhibitory results support the identical patterns of toxicity against *E. coli*-336 > MSSA-2 > MRSA-1, which is evident with well diffusion data above. CLE-CuONPs maximal toxicity to *E. coli*-336 can be related to its less rigid cell wall, containing 3 to 20 fold less peptidoglycans in Gram negative bacteria [48]. The difference in susceptibilities among the MSSA-2 and MRSA-1 was possibly due to the differences in their cell physiology, metabolism, and the microenvironment which renders the degree surface contact between NPs and the bacteria [9]. In order to validate the CLE-CuONPs induced antibacterial activities, the degree of cellular damage was compared directly with the treated and untreated bacterial cells under SEM. *E. coli*-336 cells showed cellular damage and loss of the native rod shape structure (Figure 7A,B). Similarly, CLE-CuONPs treated MRSA-1 cells show pits and cavities formation. The native spherical shape was also lost by the interaction between NPs and the bacterial cells (Figure 7C,D).

#### 3.5.3. CLE-CuONPs Internalization in Bacterial Cells

**Figure 8:**
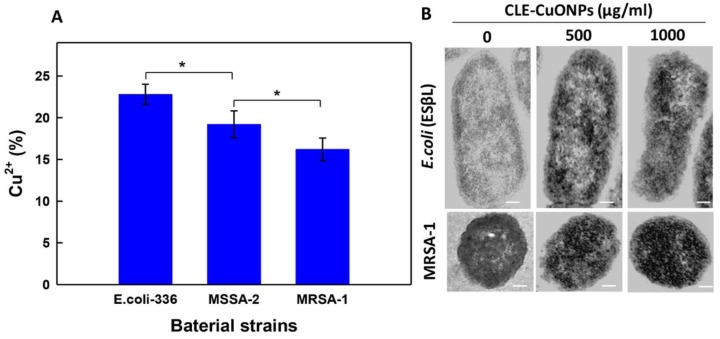
AAS detection of intracellular accumulation of Cu^2+^. (**A**) shows intracellular accumulation of Cu^2+^ in *E. coli*-336, MSSA-2 and MRSA-1. Asterisks represent significance at *p < 0.05*, while error bars represent SD of triplicate samples; (**B**) demonstrate the TEM micrographs of *E. coli*-336 and MRSA-1 exposed to CLE-CuONPs (500–1000 µg/mL) indicating the intracellular NPs accumulation, and cellular damage. Scale bar = 200 nm.

The AAS results revealed 22.80 ± 2.6%, 19. 2 ± 4.2%, and 16.2 ± 3.6% accumulation of intracellular Cu^2+^ in *E. coli*-336, MSSA-2 and MRSA-1 when exposed to CLE-CuONPs (1000 µg/mL) (Figure 8A). The order of intracellular Cu^2+^ accumulation determined as *E. coli*-336 > MSSA-2 > MRSA-1. These differences primarily relate to the structural differences between the cell wall of Gram-positive and Gram-negative bacteria. Some auxiliary factors like bacterial growth rate, expression of stress-response genes, and stability of NPs at different pH play a crucial role. In addition, the enzymatic degradation within biofilm matrix, NPs electrostatic properties, corona formation, and metal dissolution play a viable role in evaluating the sensitivity or resistance against NPs [9]. TEM analysis of CLE-CuONPs treated *E. coli*-336 and MRSA-1 cells clearly demonstrate a significant amount of NPs internalization (Figure 8B). However, bacterial cells are not known to possess endocytosis mechanism to uptake extracellular entities. Therefore, CLE-CuONPs can be speculated to enter the cells by causing damage in the intact cell membrane, which may lead to the cytoplasmic leakage [49].

### 3.6. Inhibition in Biofilm Formation

**Figure 9:**
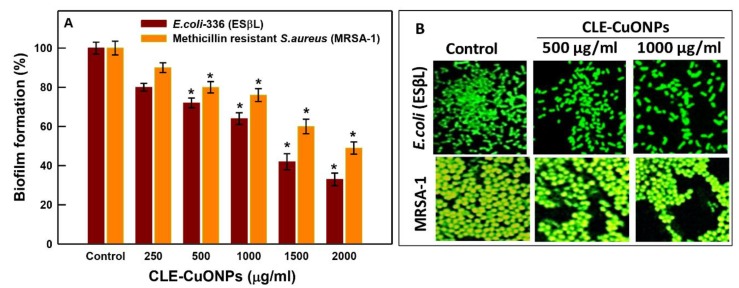
CLE-CuONPs induced antibiofilm activities in clinical isolates. (**A**) quantitative estimation of CLE-CuONPs showing inhibition of bacterial biofilm formation. The error bars represent mean ± SD of two independent experiments done in triplicates. * *p* < 0.05 vs. control; (**B**) qualitative analysis showing reduction in the biofilm formation by clinical isolates after CLE-CuONPs exposure analyzed by CLSM.

A concentration depended biofilm inhibition was recorded against the planktonic growth of cells. Compared to the untreated control (100% biofilm formation), *E. coli*-336 exposure with CLE-CuONPs suspensions (250 to 2000 µg/mL) established 80.0 ± 2.0 to 33.0 ± 3.2% biofilm formation on the bare surface of micro-wells (Figure 9A). Under the identical conditions, MRSA-1 shows 90.0 ± 2.5 to 49.0 ± 3.1% biofilm formation. CLE-CuONPs exhibit more effective antibiofilm activity against *E. coli*-336. The observed trend is in line with the greater antibacterial effects against Gram negative isolate, as compared to the Gram positive bacterial isolates. These antibacterial activities are in good agreement with the previously reported antibacterial efficacy of CuONPs [9]. The qualitative CLSM data on biofilm density (Figure 9B) correspond well with the quantitative data (Figure 9A). Increasing concentrations of CLE-CuONPs reduces the biofilm formation in *E. coli*-336 and MRSA-1, as compared to the control cells. A possible explanation of the observed effects can be surmised with the size dependent dissolution of CuONPs, and subsequent release of Cu^2+^ in the microenvironment of bacterial cells. The dissolution of Cu^2+^ in the cytoplasm after internalization NPs may assist the biomolecular interaction of Cu^2+^ with DNA. These events may trigger the repeated redox reaction chain to generate reactive oxygen species leading to the membrane damage and bacterial cell death by oxidative stress [50].

## 4. Conclusions

This study demonstrates a simple, eco-friendly biosynthesis method of esters functionalized CLE-CuONPs using an aqueous extract of CLE leaves. Comparative GC-MS analysis of CLE-alone and CLE-CuONPs provides substantial evidence that the CLE esters such as di-propyleneglicol diacrylate, α-monoolein, and iso-octyl phthalate played a crucial role in Cu^2+^ or Cu^3+^ reduction, and surface functionalization. Comparative FTIR analyses reflect the spectral signature of auxiliary bio-actives of CLE such as proteins, sugars and polyphenols in association of CuONPs. Importantly, this study also shed light on the bio-actives esters as an efficient reducing, capping, and stabilizing agents at varying pH conditions (4.0 to 12.0). Particularly, under the harsh environmental pH, the CLE-CuONPs can be speculated to act as a stable antimicrobial agent. A significant interaction and intracellular uptake of CLE-CuONPs in biofilm producing *E. coli*-336 and MSRA-1 was validated. CLSM results demonstrate the greater antibiofilm activities in *E. coli* followed by *S. aureus* owing to the differences in the cell wall compositions. Overall, our data unequivocally indicate that CLE-CuONPs may be utilized as a safer alternative against prominent MDR and biofilm infections in the biomedical settings.

## Figures and Tables

**Table 1:** GC-MS analysis of CLE leaf extract.

Peaks (P)	Retention Time (RT)	Name of Compound	Mol. Wt.	Mol. Formula	Peak Area (%)	Structure
1	29.297	Dipropyleneglicol diacrylate	242.26	C_12_H_18_O_5_	13.29	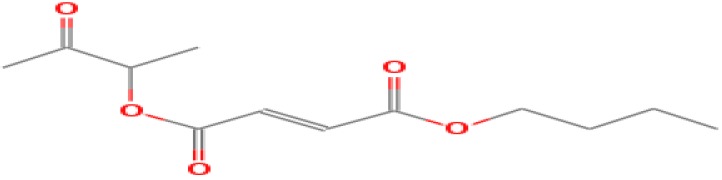
2	31.506	1-Hexadecanol	242.44	C_16_H_34_O	1.383	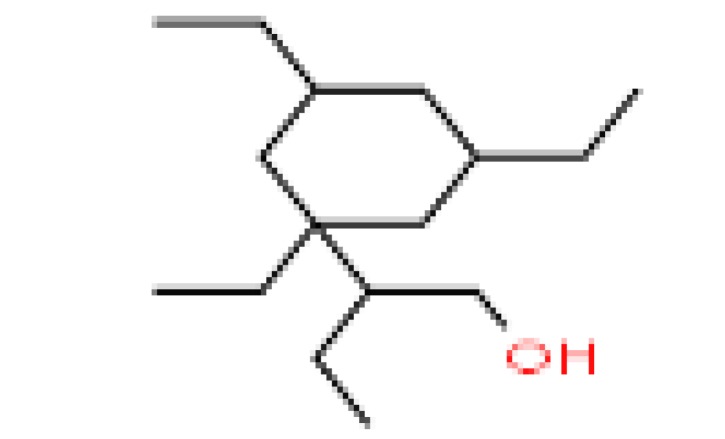
3	35.023	n-Hexadecenoic acid	256.42	C_16_H_32_O_2_	1.042	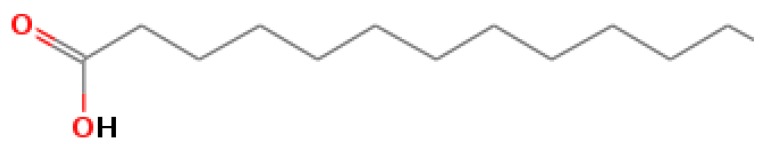
4	35.616	2-Tetradecanol	214.38	C_14_H_30_O	0.972	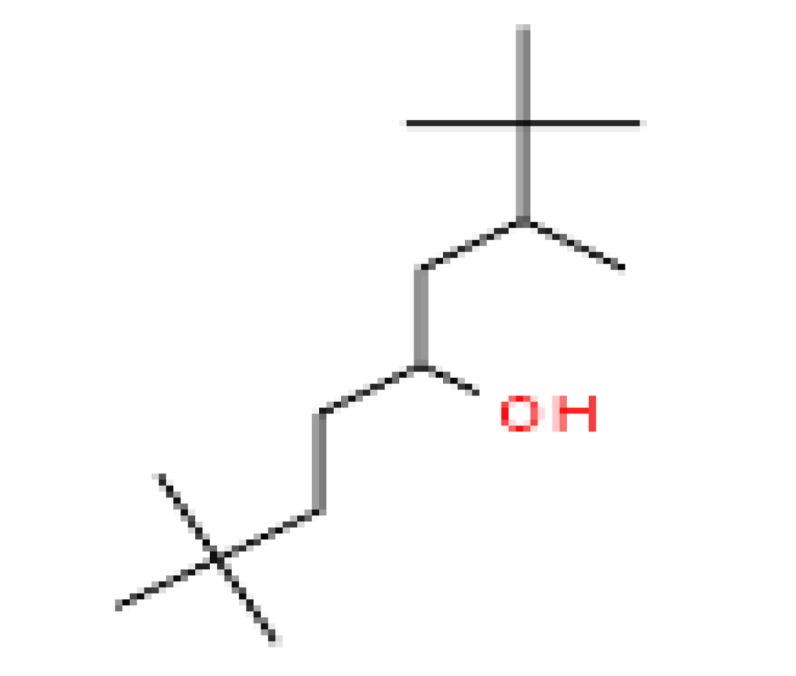
5	36.04	Dodecyl 3-mercaptopropanoate	274.46	C_15_H_30_O_2_S	1.264	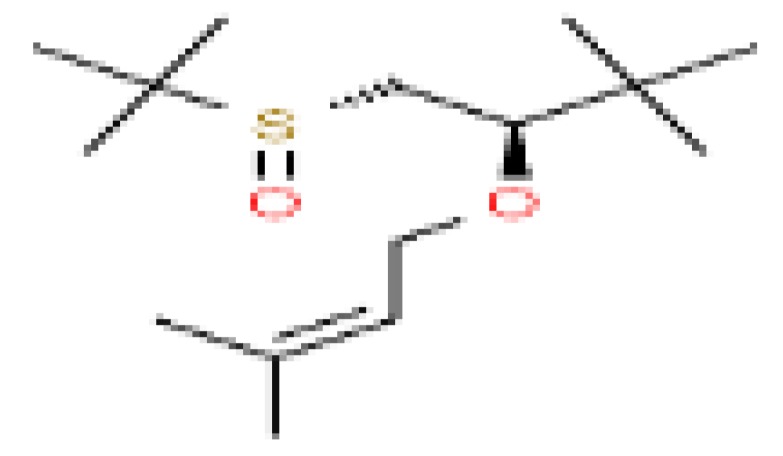
6	37.621	α,γ-Dipalmitin	296.48	C_19_H_36_O_2_	1.886	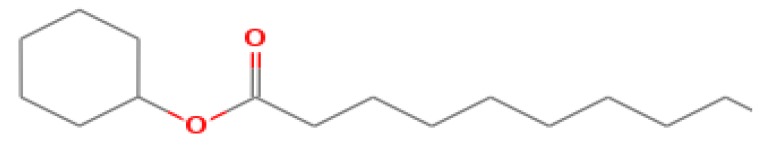
7	38.426	13-Octadecenoic acid	282.46	C_18_H_34_O_2_	6.217	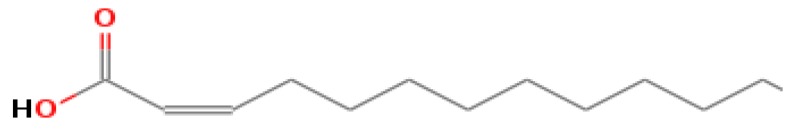
8	39.982	Glyceryl 1,2-dipalmitate	568.91	C_35_H_68_O_5_	2.665	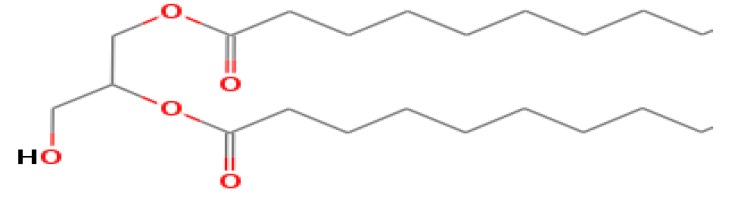
9	40.258	Glyceryl 1,2-dipalmitate	568.91	C_35_H_68_O_5_	5.058	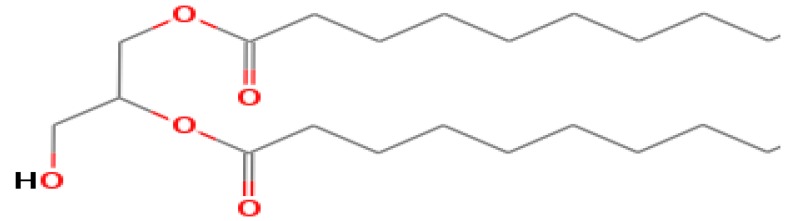
10	41.119	Glyceryl 1,2-dipalmitate	568.91	C_35_H_68_O_5_	3.903	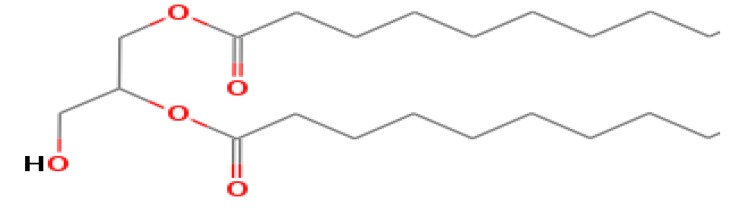
11	42.944	α-Monoolein	356.53	C_21_H_40_O_4_	16.534	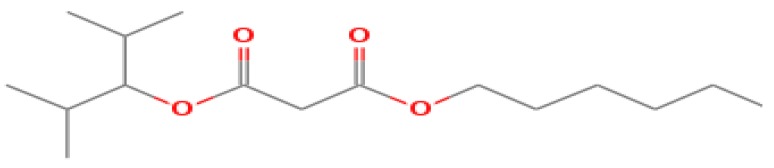
12	43.22	α-Monoolein	356.53	C_21_H_40_O_4_	21.603	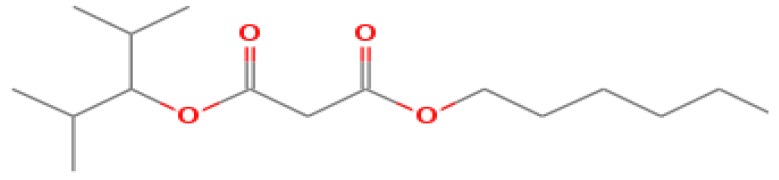
13	43.98	9, 11 Octadecadienoic acid butyl ester	336.55	C_22_H_40_O_2_	2.785	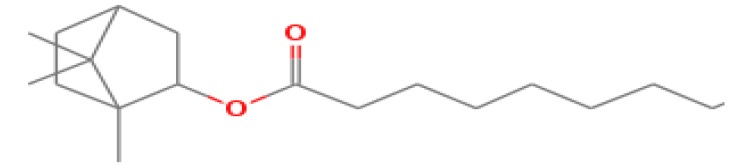
14	44.081	α-Monoolein	356.53	C_21_H_40_O_4_	18.856	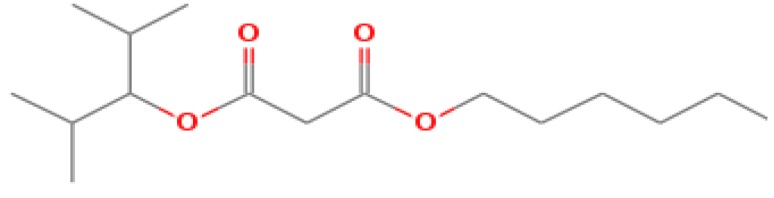
15	44.957	Isooctyl phthalate	390.55	C_24_H_38_O_4_	2.541	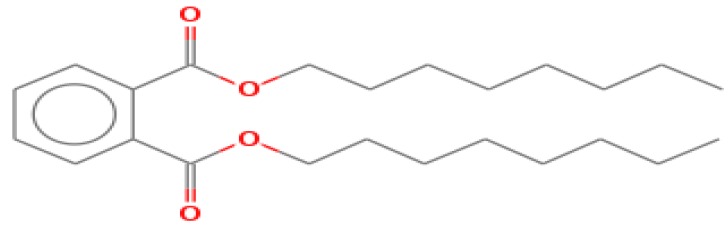

**Table 2:** GC-MS analysis of CLE-CuONPs.

Peaks (P)	Retention Time (RT)	Name of Compound	Mol. Wt.	Mol. Formula	Peak Area (%)	Structure
1	29.295	Dipropyleneglicol diacrylate	242.26	C_12_H_18_O_5_	62.315	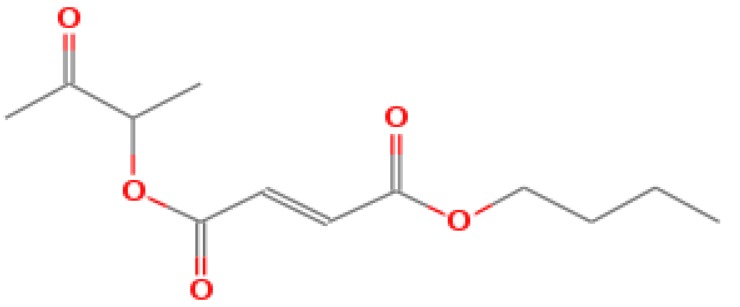
2	44.079	α-Monoolein	356.53	C_21_H_40_O_4_	37.685	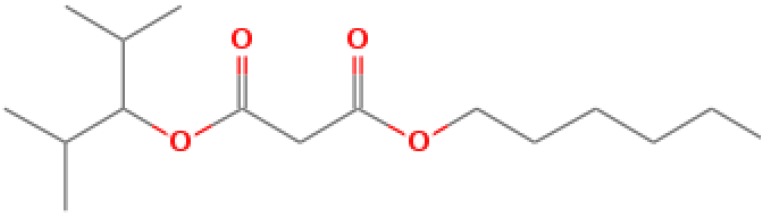
3	44.953	Isooctyl phthalate	390.55	C_24_H_38_O_4_	10.578	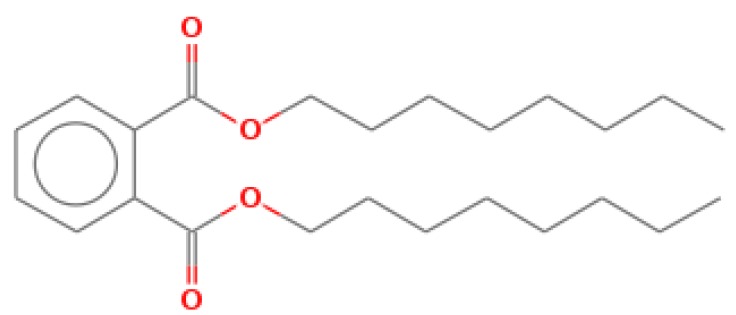

**Table 3:** MIC and MBC of clinical isolates exposed to CuONPs.

Bacterial Strain	CLE-CuONPs (µg/mL)	
	MIC	MBC
*E. coli* (*ESβL*)-336	500	1500
*S. aureus* (MSSA-2)	1000	2000
*S. aureus* (MRSA-1)	1500	2500

## Supplementary Materials

The following are available online at https://www.mdpi.com/2218-273X/10/2/169/s1, Table S1: FTIR analysis of CLE and CLE-CuONPs.
