# Supplementary material for: Cymbopogon Citratus Functionalized Green Synthesis of CuO-Nanoparticles: Novel Prospects as Antibacterial and Antibiofilm Agents
Source: Biomolecules. 2020 Jan 22;10(2):169. doi: 10.3390/biom10020169 (PMC7072505; doi:10.3390/biom10020169)
Supplement: Supplementary file 1 [file biomolecules-10-00169-s001.pdf]

GC-MS analysis of *Cymbopogon citratus* bio-active ester functionalized CuO-nanoparticles and their antibacterial and antibiofilm activities

Table S1. FTIR analysis of CLE and CLE-CuONPs

S. No.	Bond stretching (cm^{-1})		Bond assignments	Ref.
	CLE	CLE-CuONPs		
1.	3437	3572, 3488	Bonded hydroxyl (–OH) or amine groups (–NH) stretching	Chiguvare et al., 2016
2.	2845	2926	CHO vibrations	Satapathy et al., 2017
3.	1630	1628	-C=O stretch of ester and amide group	Narasaiah et al., 2017
4.	790, 692	1152, 1113, 1080, 987, 886, 801, 641, 611	-CN stretching of amine and -C-O-C group	Kumari et al., 2015
5.	-	520	Vibrations of CuONPs	Sankar et al., 2014
6.	-	491	Vibration of Cu-O bond	Sankar et al., 2014

Supplementary References:

1. Chiguvare, H., Oyedeji, O.O. , Matewu, R., Aremu, O., Oyemitan, I.A., Oyedeji, A.O., Nkeh-Chungag, B.N., Songca, S.P., Mohan, S., Oluwafemi, O.S. Synthesis of silver nanoparticles using Buchu plant extracts and their analgesic properties, Mol. **2016,** *21* 774-777.
2. Kumari, M.M. Jacob, J., Philip, D. Green synthesis and applications of Au–Ag bimetallic nanoparticles, Spectrochim. Acta Mol. Biomol. Spectrosc. **2015,** *137,* 185–192.
3. Narasaiah, P.; Mandal, B.K.; Sarada, N. Biosynthesis of copper oxide nanoparticles from *Drypetes sepiaria* leaf extract and their catalytic activity to dye degradation. In Proceedings of Materials Science and Engineering Conference Series; **2017,** p. 022012.
4. Sankar, R., Maheswari, R., Karthik, S., Shivashangari K.S., Ravikumar, V. Anticancer activity of *Ficus religiosa* engineered copper oxide nanoparticles, Mat. Sci. Engg. C **2014,** *44,* 234–239.
5. Satapathy, S., Paikaray, S., Thirunavoukkarasu, M., Panda, C.R., Subbudhi. E. Biosynthesis and characterization of silver nanoparticles derived from marine bivalve *Donax cuneatus* (Linnaeus) and assessment of its antimicrobial potential, Inorg. Nano-Met. Chem. **2017,** *47* 1044-1048.
